# Laser Ablation of Pilonidal Sinus in a Patient With Hemophilia Type A: A Case Report and Review of the Literature

**DOI:** 10.7759/cureus.59092

**Published:** 2024-04-26

**Authors:** Mohammed Basendowah, Zainab A Alkhalifah, Sarah Alasbli, Sedrah Hanbazaza, Mohammed Albladi

**Affiliations:** 1 Surgery, King Abdulaziz University Hospital, Jeddah, SAU; 2 Medical School, Faculty of Medicine, King Abdulaziz University, Jeddah, SAU; 3 Surgery, International Medical Center, Jeddah, SAU

**Keywords:** laser ablation, pilonidal sinus, pilonidal sinus treatment, new technique in pilonidal sinus surgery, infected pilonidal sinus, pilonidal sinus surgery, hemophilia-a

## Abstract

Pilonidal sinus (PNS) is a common occurrence in young men. It is often caused by blockage of the sebaceous glands in the hair follicles in the sacrococcygeal area. Hemophilia type A is a hemorrhagic disorder caused by a deficiency of factor VIII. It presents with excessive bleeding, either spontaneously or secondary to trauma. The mainstay of treatment for PNS is often excision of the sinus; however, recently, laser ablation has started to be commonly used. In this article, we present a case of a young man with hemophilia A presenting with recurrent PNS successfully managed with laser ablation with no complications.

## Introduction

Pilonidal sinus (PNS) is common in young men [1], affecting roughly 26 per 100,000 of the population [2]. Although the exact etiology is unknown, enlargement of hair follicles in the sacrococcygeal area due to hormonal changes and blockage of the pilosebaceous glands may be the cause [2]. Moreover, recurrent buttock-crease infections may evolve into chronic fistulas. Pilonidal sinus can present as a chronic discharging sinus tract or as an acute abscess [3].

Recurrent abscesses can occur, but long-term remissions are common [4]. The diagnosis can be made clinically since the appearance of pits in the natal cleft is very common [4]. The pits are easy to overlook during abscess formation due to swelling, but they are usually apparent two to three weeks after the abscess is opened or perforated [4]. In general, diagnostic tests such as computed tomography (CT), magnetic resonance imaging (MRI), endoscopy, and ultrasound are not recommended unless it is difficult to distinguish the finding from neoplasia, cystic formations, or Crohn's disease [4]. Another element of the differential diagnosis is hidradenitis suppurativa [4]. The management of an acute pilonidal abscess requires a simple incision rather than a full excision [4].

Although surgery and tissue excision are the mainstay of treatment, laser ablation has recently been used as a new method [1, 5]. Laser ablation uses a light source with laser waves that permanently destroy the hair follicles inside and cause fibrosis in the tract [1, 5]. However, studies on the efficacy of laser excision of the PNS in patients with hemophilia are lacking.

Hemophilia A is a bleeding disorder characterized by a deficiency or dysfunction of clotting factor VIII [6]. Hemophilia type A is the most common form of hemophilia and has a prevalence of one in 5,000 male births as it is an X-linked recessive trait [7]. This deficiency leads to a prolonged bleeding tendency and an increased risk of bleeding episodes, both spontaneously and following injuries or procedures [6]. Its symptoms vary depending on whether the condition is mild, moderate, or severe. The main treatment for hemophilia A is concentrated factor VIII products [6].

Replacement therapy is typically not necessary for patients with mild to severe hemophilia A unless they are about to have surgery or are experiencing a bleeding episode, as presented in this case [6]. Patients with severe hemophilia A are frequently treated with frequent factor replacement therapy [6]. A monoclonal antibody called emicizumab, which replaces factor VIII's normal function, is another potential therapy option [6].

In this study, we will present the case of a young man with hemophilia who presented with PNS and was successfully treated with laser ablation. 

## Case presentation

We present a case of a 15-year-old boy with hemophilia A who requires factor VIII transfusion upon bleeding or before invasive procedures, such as dental or surgical procedures. He first presented with sacrococcygeal pain at another facility, where he was administered antibiotics and discharged. Thereafter, the patient experienced recurrent episodes of PNS infection that were managed with antibiotics. In addition, he had external hemorrhoids, which bled once and were managed with factor VIII transfusions at another facility. Three months after the initial presentation, the patient reported to our outpatient clinic with no signs or symptoms of PNS infection and was electively admitted to our tertiary institution for laser excision of the PNS.

Back during his presentation, there was discharge from the PNS sites, and two sinuses developed. Additionally, as the patient was a hemophilia A patient, no bedside management or invasive procedure was attempted in order not to cause excessive bleeding for this high-risk patient. The diagnosis of the PNS was done clinically, and no imaging was attempted to further confirm this clinical diagnosis.

Upon presentation to our center, laser excision of the PNS was selected because it is less invasive and is associated with a lower risk of bleeding.

Preoperative hematological findings are included in Table 1.

**Table 1:** Preoperative hematological lab findings

Lab	Results	Reference range
Prothrombin level	18.8 sec	10 – 13 sec
Active partial thromboplastin time	59.4 sec	21 – 35 sec
International normalized ratio	1.53	< 1.1

The hematology department, which was contacted prior to the procedure, recommended administration of factor VIII 2,000 U 30 minutes before the operation, 2,000 U 24 hours postoperatively, and 1,000 U for three days thereafter, and 500 mg of tranexamic acid thrice daily for three to five days starting on the day of surgery.

**Figure 1:**
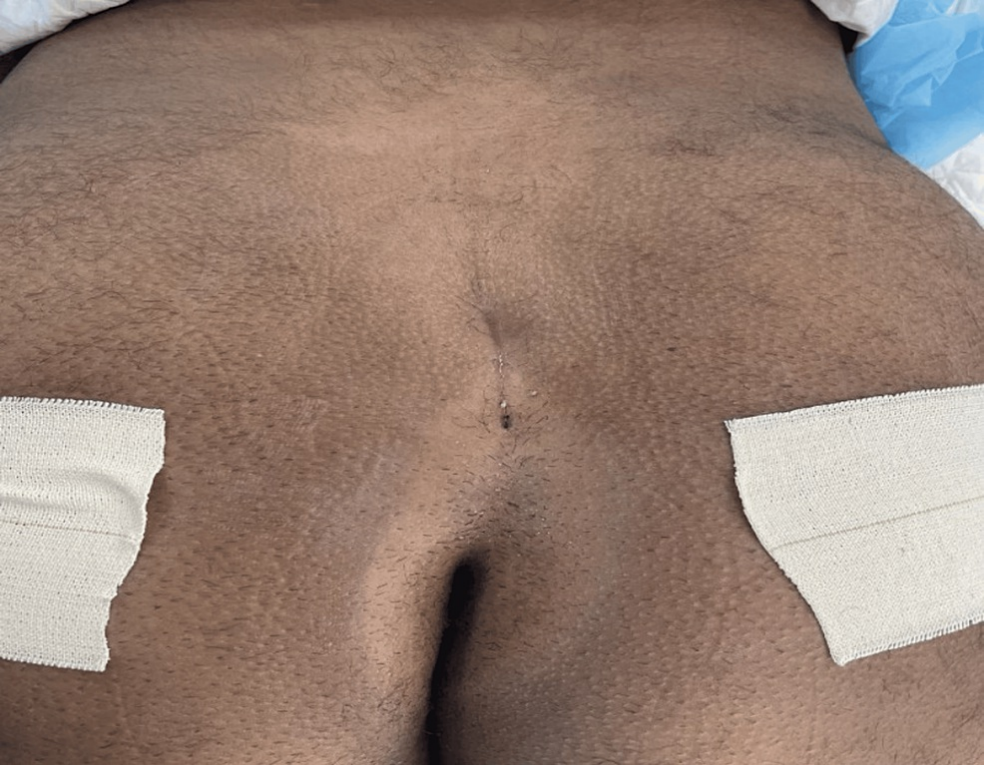
A preoperative photograph shows the back (sacrum) of the patient, which shows two sinuses, one superior and one inferior.

**Figure 2:**
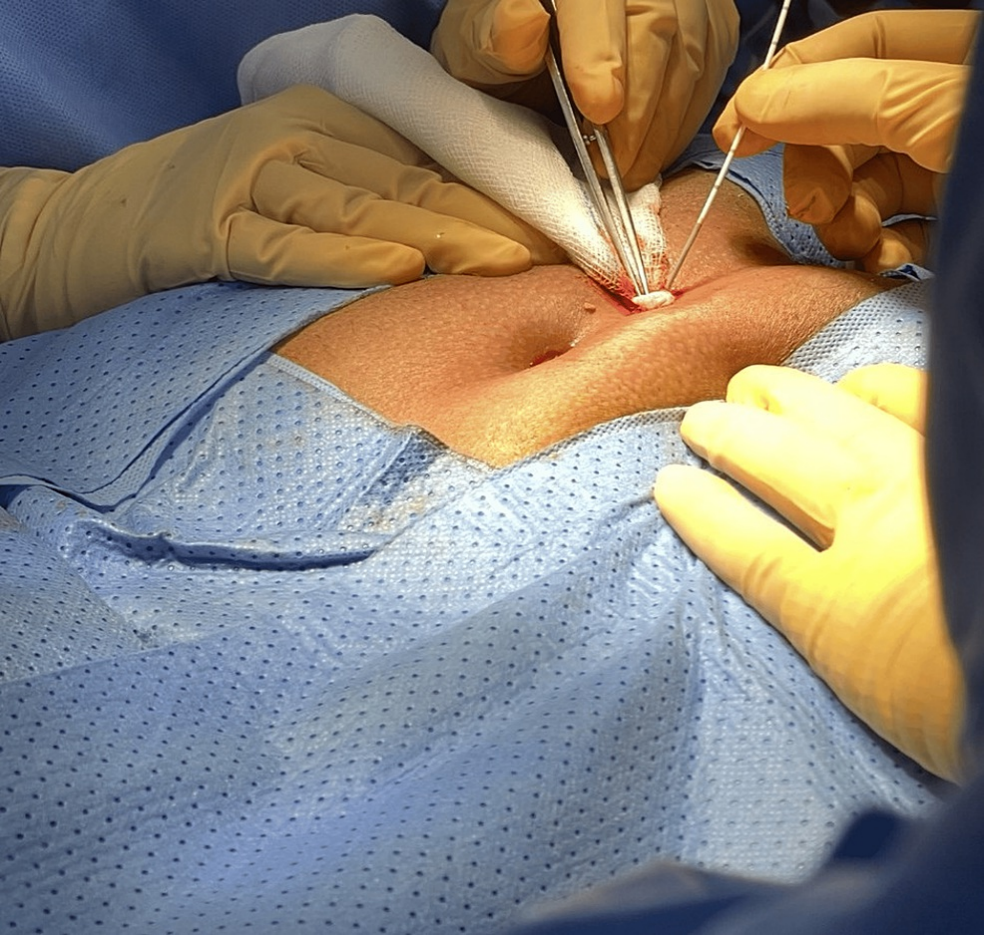
This intraoperative picture shows ablation being performed using a laser probe.

**Figure 3:**
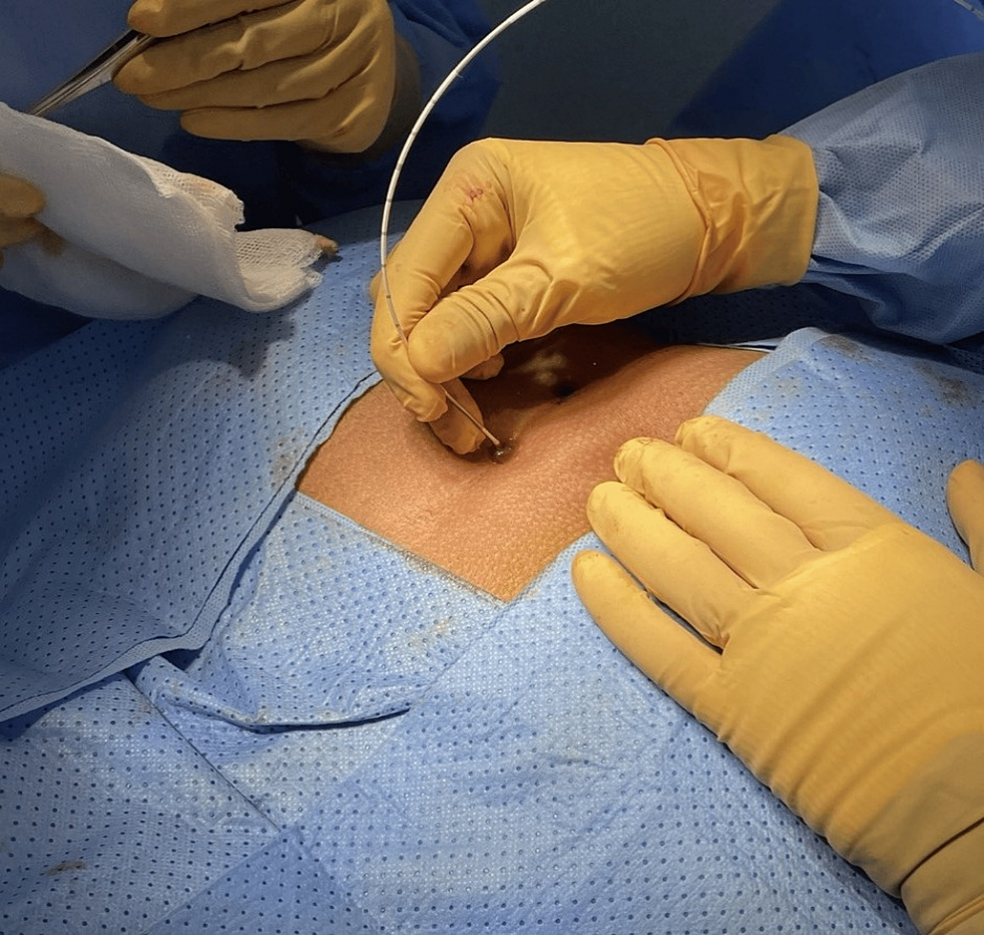
Another intraoperative picture shows ablation being performed using a laser probe.

Under general anesthesia, the patient was placed in a prone position. Draping was performed in a sterile manner, and the surgical area was cleaned with iodine. Two sinuses were observed, one containing hair and another 5 cm above it (Figure 1). Hair was removed using a curette, followed by irrigation of the sinus tracts with saline and hydrogen peroxide. Ablation of both tracts was performed using a laser probe at 8 W and 1470 J (Figures 2, 3). Hemostasis was achieved with minimal bleeding, and a regular dressing was applied. The duration of the procedure was 29 minutes, excluding the anesthesia-induction time.

**Figure 4:**
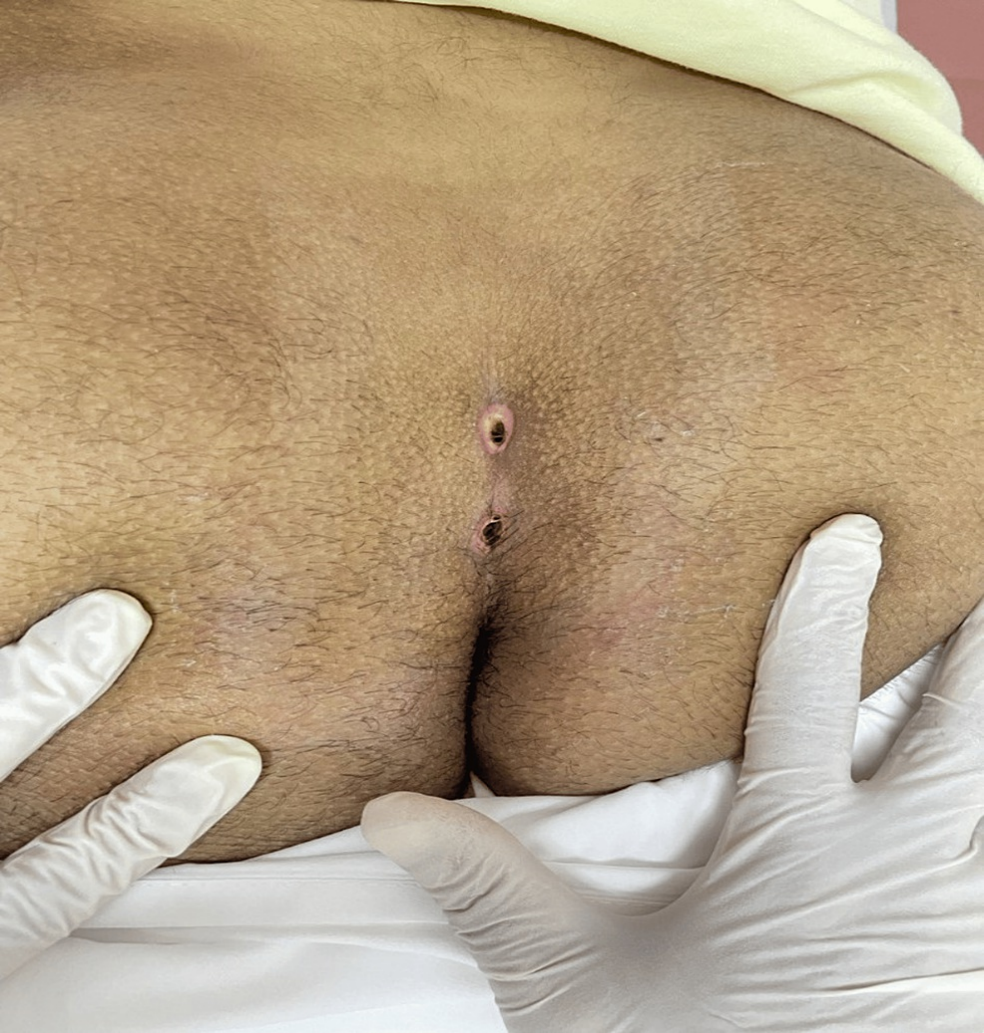
An image of the two sinuses two weeks after surgery

**Figure 5:**
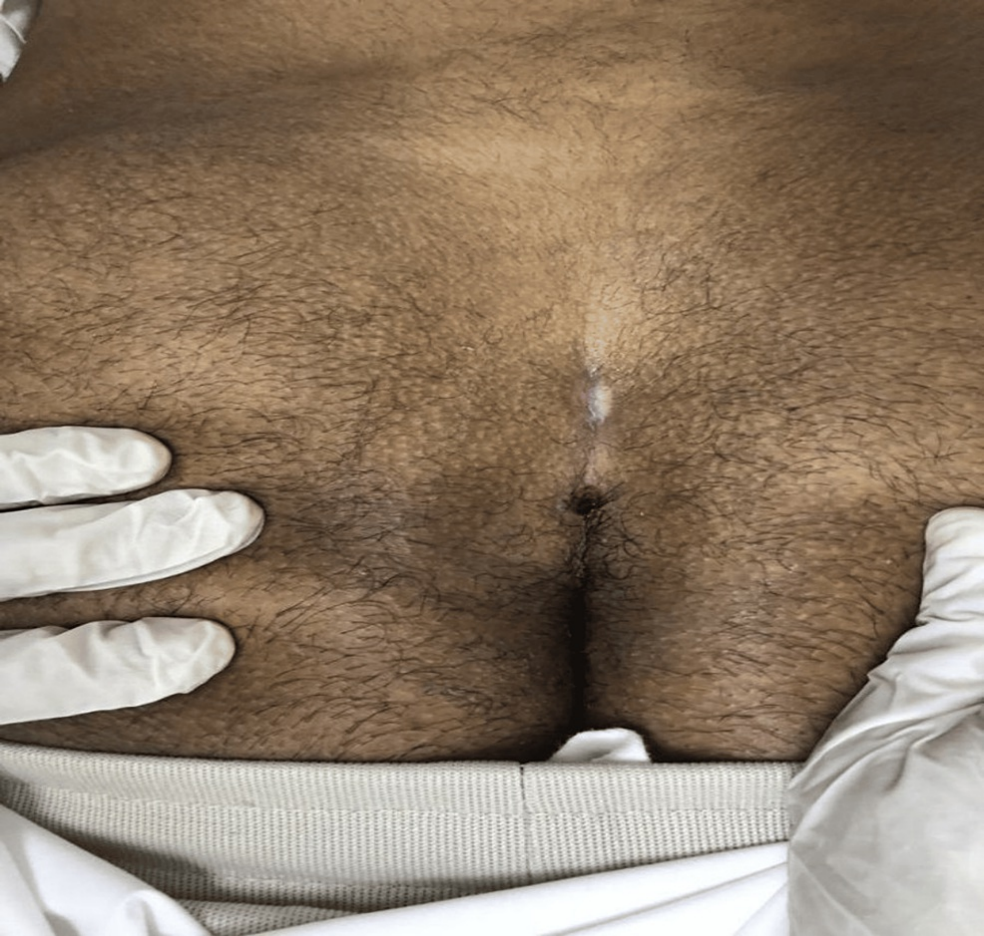
An image of the two sinuses four weeks after surgery shows the sinuses healing.

**Figure 6:**
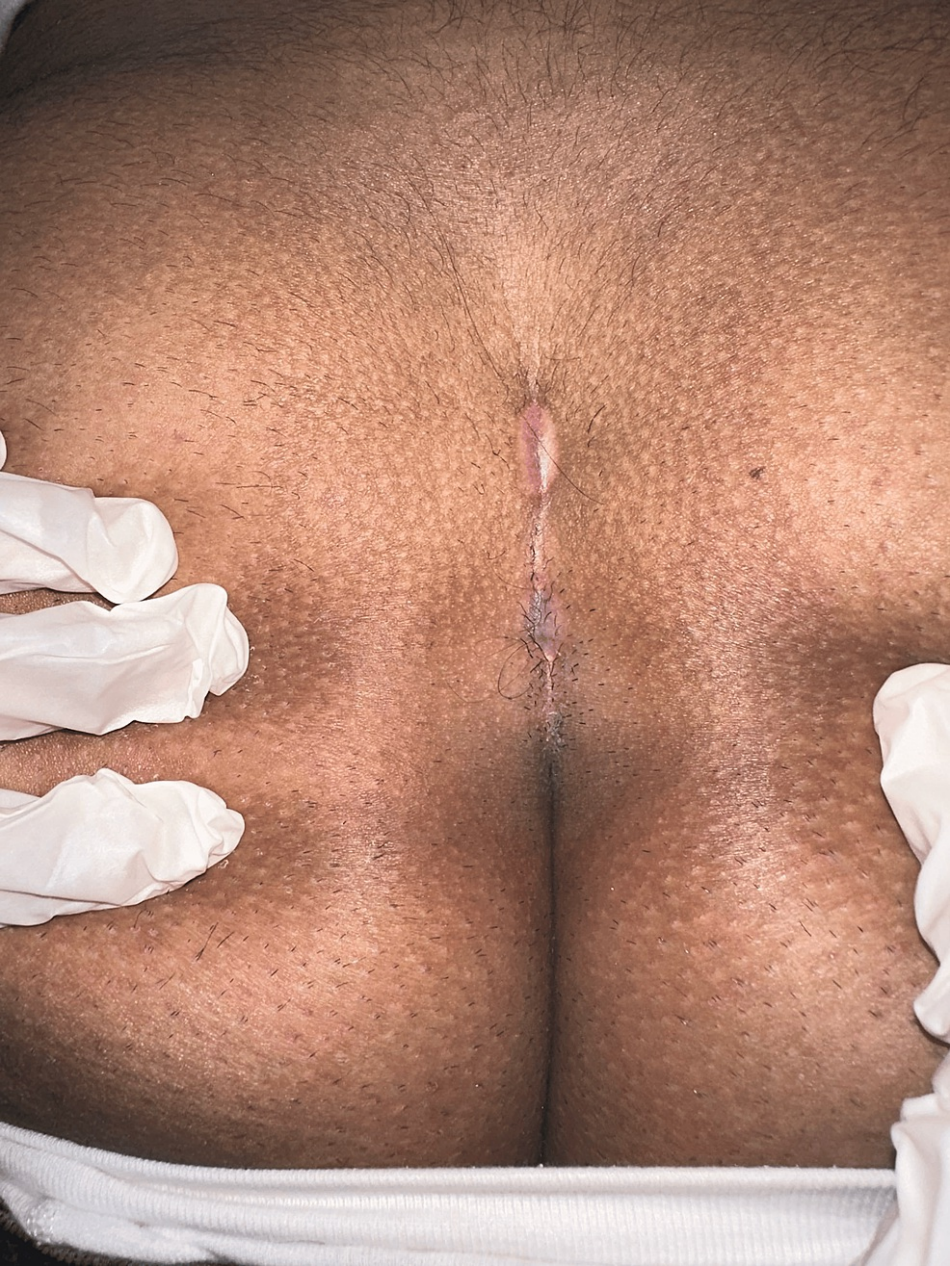
An image taken six weeks postoperatively shows complete healing with minimal scarring.

Postoperatively, the patient received factor VIII (2,000 units) and tranexamic acid as scheduled and was discharged on postoperative day two with no complications. At the outpatient follow-up one week after surgery, the patient was doing well with no complaints, and the surgery site had started to heal. Figures 4-6 show postoperative healing at different time points.

This was a successful case of laser ablation with a probe in a case of recurrent PNS in a young male who is a known case of hemophilia A. In hemophilia A patients, physicians tend to be more careful with invasive procedures as they are at high risk for bleeding. Laser ablation was especially preferred in his case, given the risk of bleeding that can be encountered with a regular surgical excision. As well, this approach was less invasive to the patient.

## Discussion

An extensive search of the PubMed database for case series, reports, and reviews on PNS in patients with hemophilia did not identify any relevant studies. The literature review revealed that laser ablation using a 1470‐nm radial diode laser fiber is effective for the treatment of PNS because of its minimally invasive nature and is associated with reduced postoperative pain, shorter healing time, and potentially lower recurrence rates compared to traditional surgical techniques. Furthermore, few studies have reported favorable outcomes with laser ablation, including improved wound healing and lower recurrence rates [8, 9].

Laser ablation of PNS avoids the need for extensive surgical incisions and tissue removal. The laser vaporizes and ablates the sinus tracts while simultaneously sealing the surrounding tissue, thereby minimizing damage and promoting faster healing. This approach results in smaller wounds, reduced postoperative pain, and improved patient satisfaction [9]. Laser energy selectively targets and destroys the sinus tracts, leaving the surrounding healthy tissues intact. This targeted approach minimizes the risk of damage to adjacent structures and reduces the likelihood of infection [10].

In addition, laser ablation results in minimal tissue trauma, and reduces postoperative pain and discomfort. Patients often experience faster wound healing, which enables them to return to their daily activities sooner. This shorter recovery period not only improves patient satisfaction but also reduces the economic burden associated with prolonged hospital stays and time off work [4].

Meanwhile, traditional surgical techniques can leave unsightly scars, which can cause distress and affect body image. Laser ablation offers improved cosmetic outcomes owing to its minimal invasiveness. The smaller incisions heal faster and with less scarring, resulting in improved aesthetics and body confidence [11].

Review of the literature

The comparative analysis done in Table 2 provides an overview of the identified studies, presenting information on study design, sample size, treatment approach, and outcomes. It includes case reports, case series, pilot studies, and reviews that evaluated laser ablation or laser therapy for PNS. The studies collectively support the use of laser ablation as a promising technique for PNS treatment.

**Table 2:** Comparative analysis of studies on laser ablation for pilonidal sinus

Author	Study Design	Sample Size	Treatment Approach	Outcomes
Landa et al., 2005 [1]	Clinical study	6	Laser epilation	The outcomes suggest that laser hair removal in the natal cleft is an effective alternative to surgical treatment for pilonidal disease.
Allam et al., 2020 [5]	Case series	20	Laser therapy	It is considered a competitive alternative to other surgical interventions for pilonidal sinus. Laser pilonidoplasty was associated with reduced pain, early resumption of work, and a decreased rate of recurrence.
Li et al., 2023 [8]	Case series	48	Laser ablation	The study suggests that this technique could be considered a first-line treatment for most patients with sacrococcygeal pilonidal disease (SPD), including those in the chronic stage and those in the acute stage without an abscess.
Williams et al., 2023 [9]	Pilot study	25	Laser ablation	The feasibility and safety of laser ablation was demonstrated.
Ganduboina et al., 2023 [4]	Review	N/A	Laser ablation	Based on the review of 44 articles obtained from PubMed, Cochrane, and Google Scholar, laser ablation procedures demonstrated good treatment efficacy in pilonidal sinus disease.

Potential limitations

While laser ablation shows promise as a minimally invasive technique for PNS treatment, it is not without limitations. The success of laser ablation relies on the accurate identification and mapping of sinus tracts, which can be challenging in complex cases. Furthermore, the procedure may not be suitable for large or deep-seated sinus tracts, necessitating alternative treatment options. In addition, long-term studies are needed to evaluate the durability and recurrence rates associated with laser ablation.

## Conclusions

We reported a case of successful laser ablation of recurrent PNS in a young male with hemophilia A. Based on our presented case of a 15-year-old boy with hemophilia A and a recurrent PNS infection, we can draw several important conclusions regarding the use of laser ablation as a treatment option. Laser ablation was proven in our study to be a successful and less invasive technique for managing PNS in a patient with hemophilia A who carried a higher risk of bleeding during invasive procedures. The case highlights the potential of laser ablation to address the unique challenges faced by patients with bleeding disorders. By minimizing tissue trauma and cauterizing blood vessels, laser ablation significantly reduces the risk of bleeding complications associated with traditional surgical techniques. In this case, the patient received factor VIII transfusion and tranexamic acid as part of a preoperative and postoperative management plan to ensure hemostasis.

Therefore, we can conclude that laser ablation is a promising, minimally invasive technique for PNS treatment. It offers several advantages, including reduced postoperative pain, shorter healing time, improved cosmetic outcomes, and potentially lower recurrence rates compared to traditional surgical techniques. Laser ablation can potentially revolutionize PNS treatment and improve the quality of life of affected individuals by enhancing comfort and satisfaction.

Furthermore, this case underscores the need for further research and long-term studies to evaluate the efficacy, durability, and recurrence rates associated with laser ablation. While the presented case was successful, larger studies involving patients with hemophilia A and PNS are necessary to establish the generalizability and long-term outcomes of this approach. Additionally, more comprehensive investigations are required to address the limitations of laser ablation, such as accurately identifying complex sinus tracts and determining their suitability for large or deep-seated cases.

Overall, this case study demonstrates the successful use of laser ablation in treating recurrent PNS in a young male patient with hemophilia A, highlighting the potential benefits of this approach in reducing the risk of bleeding and providing a less invasive treatment option. This case has broader implications for clinical practice, suggesting that laser ablation is a promising treatment for PNS, particularly in patients with bleeding disorders. Further research and long-term studies are warranted to fully assess the efficacy, durability, and recurrence rates associated with laser ablation, ultimately paving the way for improved outcomes and quality of life for individuals with PNS.
